# Elongated umbilical cord with four loops around neck

**DOI:** 10.11604/pamj.2021.39.252.30349

**Published:** 2021-08-19

**Authors:** Rohan Kumar Singh, Gaurav Vedprakash Mishra

**Affiliations:** 1 Department of Radiodiagnosis, Jawaharlal Nehru Medical College, Datta Meghe Institute of Medical Sciences, Sawangi (Meghe), Wardha, India

**Keywords:** Loop of cord around neck, pregnancy, elongated umbilical cord

## Image in medicine

A 32-year-old woman at 37.5 weeks of gestation (G2P1L1A0) visited the antenatal clinic at the hospital with decreased foetal movements. Her second-trimester ultrasound showed mild intrauterine growth retardation. The first baby was normal vaginal delivery with no evidence of a loop of cord around the neck or any entanglements. On Doppler ultrasonography, decreased foetal heart rate was recorded with oligohydramnios and the patient was taken for caesarean delivery. A male neonate weighing 2.3 kgs was born with four loops of umbilical cord around the neck with no knots in the cord. The baby cried immediately after birth with a good tone and activities with a normal Apgar score. On examination, the placenta was normal with no retroplacental clot. The umbilical cord was abnormally long measuring 86 cm with three vessels.

**Figure 1:**
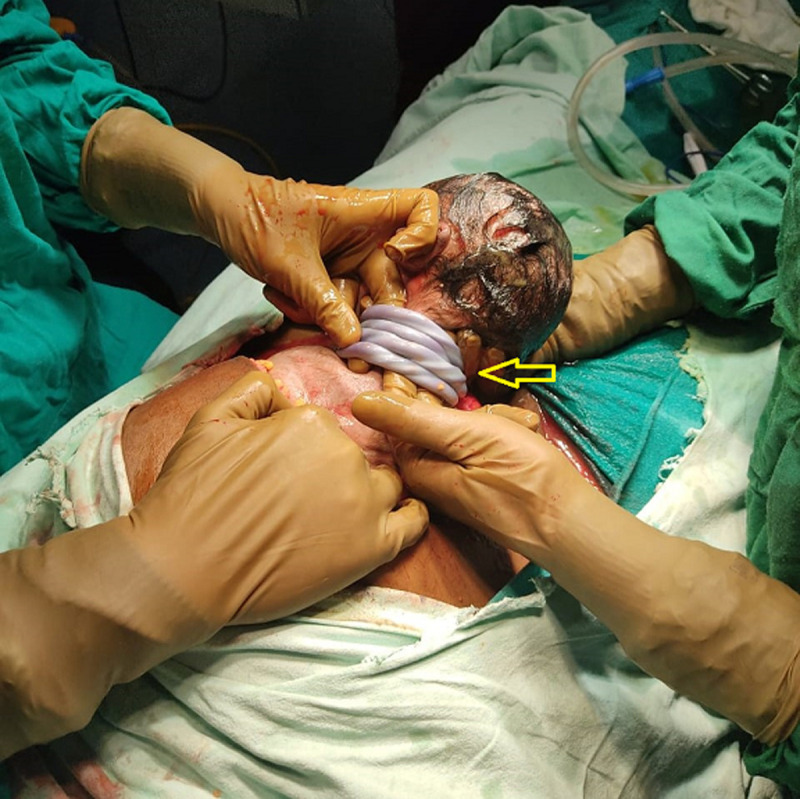
baby born with four loops of cord around the neck

